# Heme crystallization in a Chagas disease vector acts as a redox-protective mechanism to allow insect reproduction and parasite infection

**DOI:** 10.1371/journal.pntd.0006661

**Published:** 2018-07-23

**Authors:** Caroline M. Ferreira, Renata Stiebler, Francis M. Saraiva, Guilherme C. Lechuga, Ana Beatriz Walter-Nuno, Saulo C. Bourguignon, Marcelo S. Gonzalez, Patrícia Azambuja, Ana Caroline P. Gandara, Rubem F. S. Menna-Barreto, Gabriela O. Paiva-Silva, Marcia C. Paes, Marcus F. Oliveira

**Affiliations:** 1 Instituto de Bioquímica Médica Leopoldo de Meis, Universidade Federal do Rio de Janeiro, Rio de Janeiro, Rio de Janeiro, Brazil; 2 Instituto Nacional de Ciência e Tecnologia em Entomologia Molecular (INCT-EM), Rio de Janeiro, Rio de Janeiro, Brazil; 3 Instituto Oswaldo Cruz, Fiocruz, Rio de Janeiro, Rio de Janeiro, Brazil; 4 Instituto de Biologia Roberto Alcântara Gomes, Universidade do Estado do Rio de Janeiro, Rio de Janeiro, Rio de Janeiro, Brazil; 5 Departamento de Biologia Celular e Molecular, Universidade Federal Fluminense, Niterói, Rio de Janeiro, Brazil; 6 Departamento de Biologia Geral, Universidade Federal Fluminense, Niterói, Rio de Janeiro, Brazil; Instituto de Ciências Biológicas, Universidade Federal de Minas Gerais, BRAZIL

## Abstract

Heme crystallization as hemozoin represents the dominant mechanism of heme disposal in blood feeding triatomine insect vectors of the Chagas disease. The absence of drugs or vaccine for the Chagas disease causative agent, the parasite *Trypanosoma cruzi*, makes the control of vector population the best available strategy to limit disease spread. Although heme and redox homeostasis regulation is critical for both triatomine insects and *T*. *cruzi*, the physiological relevance of hemozoin for these organisms remains unknown. Here, we demonstrate that selective blockage of heme crystallization *in vivo* by the antimalarial drug quinidine, caused systemic heme overload and redox imbalance in distinct insect tissues, assessed by spectrophotometry and fluorescence microscopy. Quinidine treatment activated compensatory defensive heme-scavenging mechanisms to cope with excessive heme, as revealed by biochemical hemolymph analyses, and fat body gene expression. Importantly, egg production, oviposition, and total *T*. *cruzi* parasite counts in *R*. *prolixus* were significantly reduced by quinidine treatment. These effects were reverted by oral supplementation with the major insect antioxidant urate. Altogether, these data underscore the importance of heme crystallization as the main redox regulator for triatomine vectors, indicating the dual role of hemozoin as a protective mechanism to allow insect fertility, and *T*. *cruzi* life-cycle. Thus, targeting heme crystallization in insect vectors represents an innovative way for Chagas disease control, by reducing simultaneously triatomine reproduction and *T*. *cruzi* transmission.

## Author summary

Chagas disease is a fatal illness caused by *Trypanosoma cruzi* parasites, which are transmitted by blood sucking triatomine insect vectors. Although blood is a natural food source for these insects, its digestion releases toxic products, which poses a dietary challenge for both triatomine insects and trypanosomes. To overcome this, triatomines eliminate these toxic blood products by a unique process of heme crystallization into hemozoin that take place in their digestive tract. Here we describe that this detoxification process represents the major mechanism for redox balance control, and is necessary to allow triatomine insect reproduction, and *Trypanosoma cruzi* infection. Disruption of heme crystallization in triatomine insects thus represents a new venue for Chagas disease control, by targeting at the same time insect reproduction and parasite transmission.

## Introduction

Chagas disease (CD) is a chronic and debilitating illness caused by the protozoa *Trypanosoma cruzi* [1], afflicting about 6 million people predominantly in Latin America [2]. Despite low relative mortality of CD patients, development of chronic manifestations, such as digestive, cardiovascular and neurological disorders, pose a huge burden to the patients. *Trypanosoma cruzi* is usually transmitted to mammalians through the contact of infected feces of triatomine insects with mucosa or skin lesions [1]. Strategies designed to directly eliminate parasites in human hosts are still inefficient, making the control of the triatomine vector population the most useful method to prevent CD dissemination.

*Trypanosoma cruzi* replicates and differentiates in the digestive tract of triatomine insects, which ingest about five times their own weight on vertebrate blood to meet their energy demands. As a result, huge amounts of toxic “free” heme are released in triatomine digestive tract [3]. On one hand, *Trypanosoma cruzi* lacks a functional heme biosynthetic pathway [4], making this parasite strictly dependent on heme to support proliferation mediated by specific redox-dependent signaling pathways [5–8]. On the other hand, reactive oxygen species (ROS), propagated by "free" heme affects *T*. *cruzi* differentiation to infective trypomastigote forms [7]. This suggests that *T*. *cruzi* adapted to a unique environment in the triatomine midgut where "free" heme levels are tightly regulated. Indeed, excessive levels of “free” heme are deleterious, not only to trypanosomes [7–9], but also to triatomine vectors [3,10,11].

The endogenous release of massive amounts of heme in the triatomine midgut under normal circumstances is counteracted by a very effective array of protective adaptations to deal with this toxic molecule [3,12,13]. Heme crystallization into Hemozoin (Hz) represents the main protective mechanism against heme toxicity in many organisms that digest hemoglobin, such as malaria parasites, *Schistosoma* worms, and triatomine insects [14–16]. Indeed, heme crystallization is a very efficient mechanism for heme disposal in these organisms, accounting for about 95% of the iron derived from blood intake [14,15]. Hz crystals are produced by the interaction of "free" heme with amphiphilic structures, including food vacuole membranes in malaria parasites, extracellular lipid droplets in *Schistosoma* gut, and phospholipid membranes in triatomine insects [10,17–19]. Regardless of the organism model, aminoquinoline drugs can either form stable complexes with heme, and directly interact with Hz crystals [20–22] which ultimately impair Hz formation, building up heme and oxidized products levels [10,23–25]. As the cytotoxic properties of "free" heme were extensively explored [9,11,26,27], the mechanism for the antimalarial effects of aminoquinolines are currently explained through a redox imbalance process, and the resultant molecular damage from impaired Hz formation [28].

Although the regulation of heme homeostasis is critical for both triatomine vectors [3,12,13], and *T*. *cruzi* parasites [4–9], the physiological significance of Hz for these organisms remains unknown. We hypothesized that pharmacological blockage of Hz formation in the triatomine insect *Rhodnius prolixus* might dysregulate the heme/redox homeostasis and disrupt vector/parasite physiology ultimately, with potential effects in CD transmission. To fill this gap of knowledge, we have investigated here the biochemical and physiological effects of the aminoquinoline drug quinidine (QND) to the triatomine insect *Rhodnius prolixus*. Selective blockage of heme crystallization *in vivo* by QND caused a systemic heme overload and redox imbalance in distinct insect tissues. QND treatment activated compensatory defensive heme-scavenging mechanisms to cope with excessive heme. Importantly, egg production and laying, and total *T*. *cruzi* parasite counts in *R*. *prolixus* were significantly reduced by QND treatment. The physiological effects of QND were partially reversed by the major insect antioxidant urate. Taken together, these results indicate that heme crystallization represents the prime redox regulator for triatomine vectors, highlighting the dual role of Hz as a protective mechanism to allow insect reproduction, and *T*. *cruzi* infection, opening new possibilities for effective CD control.

## Results

### Impairment of Hz formation promotes midgut redox imbalance

**Fig 1:**
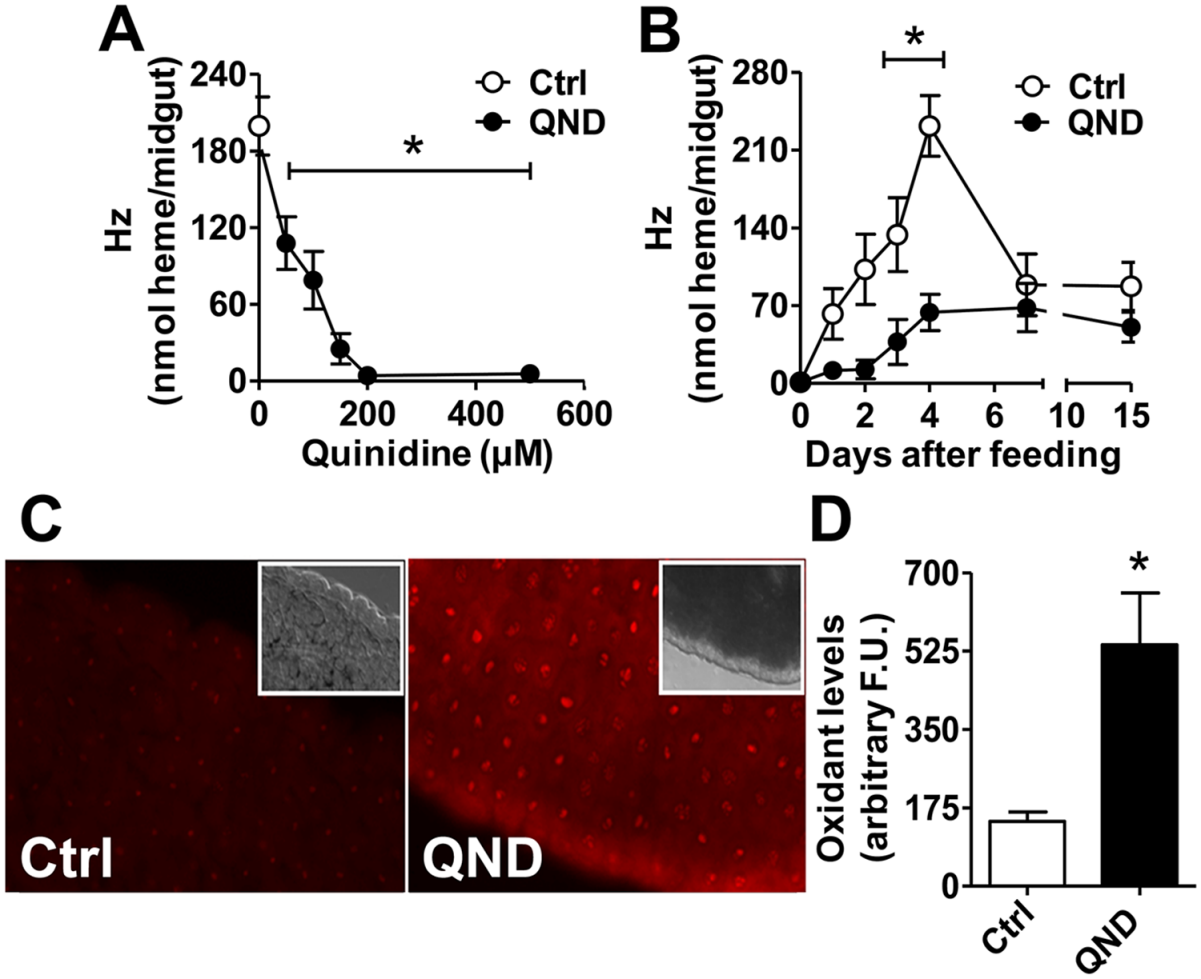
Quinidine inhibits heme crystallization *in vivo* and causes redox imbalance in triatomine posterior midgut. Insects were fed with blood (Control, Ctrl) or blood supplemented with quinidine (QND). **(A)** Posterior midgut Hz content four days after feeding. Ctrl: n = 8; QND: n≥3. Comparisons between groups were done by one-way ANOVA and *a posteriori* Tukey’s tests (*p<0.05 relative to Ctrl). **(B)** Hz content in posterior midgut along 15 days after blood meal. Ctrl: n = 8; 100 μM QND: n≥3. Comparisons between groups were done by two-way ANOVA and *a posteriori* Bonferroni’s tests (*B*; *p<0.05 relative to Ctrl). **(C)** Intracellular oxidants levels assessed by fluorescence microscopy of dihydroethidium (DHE) stained posterior midguts four days after feeding (400x magnification). Insets are bright field images of the same midgut regions. **(D)** The average DHE fluorescence intensity in the posterior midgut of insects. Ctrl: n = 24; 100 μM QND: n = 35). Comparisons between groups were done by Mann Whitney’s test (D; *p<0.0005 relative to Ctrl). All data are expressed as mean ± S.E.M. and experiments were conducted using adult insects only from IBqM colony.

To establish optimal conditions for oral administration of QND, insect survival, blood intake and digestion were assessed using individuals from two distinct colonies (Federal University of Rio de Janeiro, hereafter named "IBqM", and Fundação Oswaldo Cruz-Rio de Janeiro, hereafter named "Fiocruz"). We observed that dietary QND supplementation at 100 μM caused no significant effects in any of these outputs (S1 Fig). However, Hz formation *in vivo* was strikingly inhibited in the midgut of adult triatomines in a dose (Fig 1A), and time (Fig 1B and S2A Fig) dependent manner. The highest inhibitory activity of QND on heme crystallization (>70%) occurred around 4 days after blood meal in both colonies (Fig 1B and S2A Fig). Inhibitory effects of QND on Hz formation were also observed in young nymphs (S2B Fig). Since hemoglobin digestion, heme release and crystallization take place in the lumen of posterior midgut, we postulated that this tissue would be the first line of defense against “free” heme overload. Conversely, the posterior midgut would also be the most directly affected tissue by impaired Hz formation. As the pro-oxidant and toxic effects of heme are strongly reduced upon its crystallization as Hz [26], we anticipated that impaired Hz formation would increase heme-derived oxidant levels. Indeed, feeding adult insects with 100 μM QND shifted posterior midgut redox balance towards oxidation, as revealed by 3.7 folds increase in the fluorescence intensity of the oxidant-sensitive dye dihydroethidium (DHE) (Fig 1C and 1D). Therefore, selective inhibition of Hz formation *in vivo* promotes posterior midgut redox imbalance.

### Midgut cell architecture is affected by limited Hz formation

**Fig 2:**
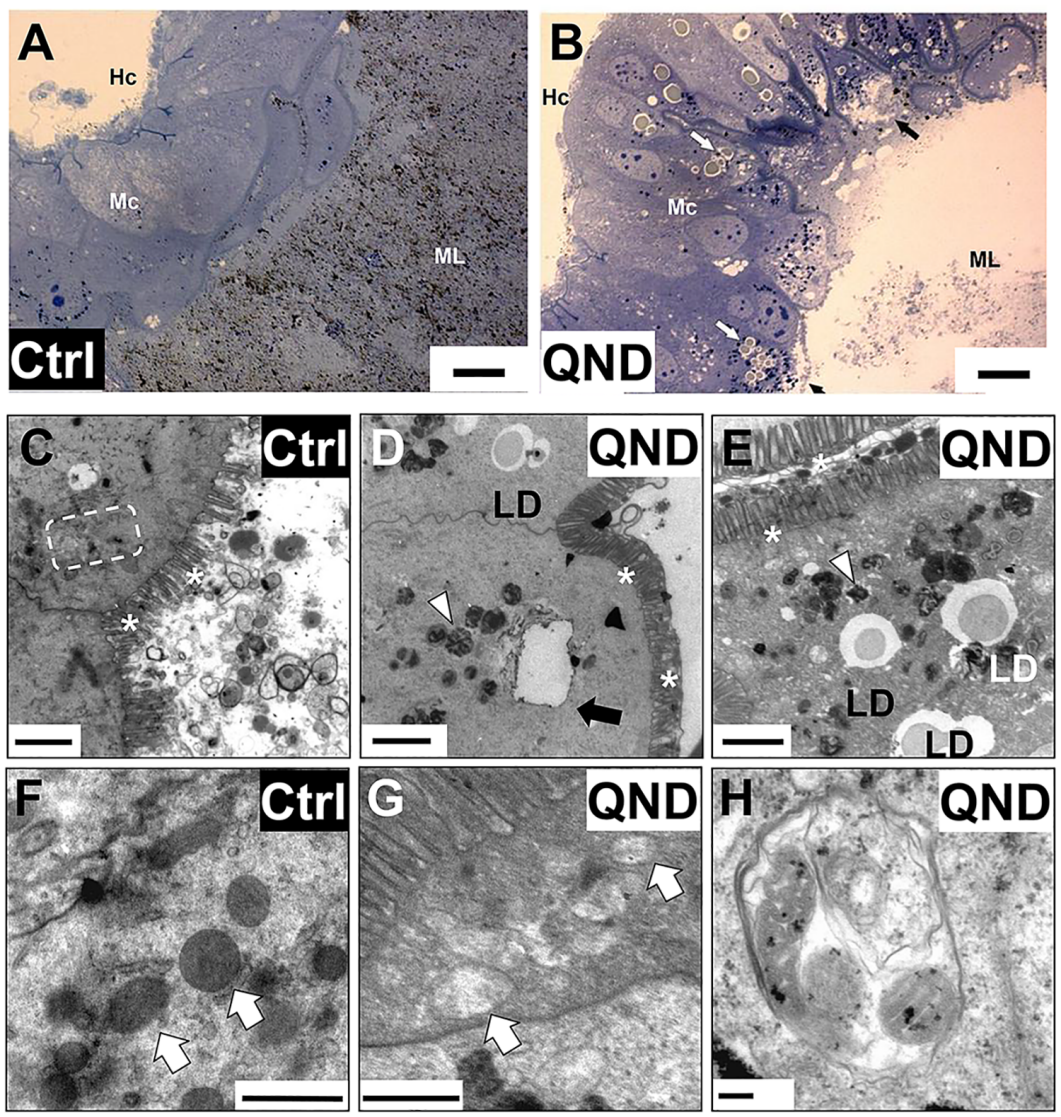
Impaired heme crystallization affects posterior midgut organization. **(A and B)** Light microscopy images of posterior midguts of adult insects fed with blood (Ctrl; **A**) and blood supplemented with 100 μM QND (**B**) from IBqM colony four days after blood meal (bars = 20 μm). Midgut lumen (ML), midgut cells (Mc), hemocoel (Hc), vacuoles (black arrows) and lipid droplets (white arrows) are indicated in the images. **(C-H)** Transmission electron microscopy images of posterior midguts from insects maintained at IBqM (**C-F**), and Fiocruz (**G,H**) colonies fed with blood (Ctrl, **C,F**), or blood + 100 μM QND (**D,E,G,H**) four days after blood meal. The general architecture of posterior midgut cells in control insects (**C,F**) includes mitochondria (white arrows), endoplasmic reticulum (dashed box) and microvilli (asterisks). In QND treated insects (**D,E,G,H**), extensive organelle disappearance contrasts with the presence of numerous electron-dense hemoxisomes/residual bodies (arrowheads), vacuoles (black arrow), and intracellular lipid droplets (LD). Electron-dense mitochondria found in the posterior midgut of control insects (**F**), contrast with swollen and washed out mitochondria from 100 μM QND treated insects (**G**). **(H)** Mitochondria inside an autophagosome. (Scale bars: C-E: 2 μm; F: 1.0 μm; G,H: 0.5 μm).

Given the higher oxidant levels resulted from impaired Hz formation in the posterior midgut, we further investigated the potential consequences to tissue and cell architecture. Histological observations of posterior midgut cross sections from QND-treated insects by light microscopy revealed that the midgut lumen was completely washed-out, with barely noticeable Hz crystals, along with extensive cytosolic vacuolization, and numerous intracellular lipid droplets in midgut cells (Fig 2A and 2B). At the cellular level, QND treatment caused severe ultrastructural changes in the posterior midgut, with remarkable disappearance of organelles resembling macroautophagy, as numerous vacuoles, lipid droplets, residual bodies, and autophagosomes were detected (Fig 2D, 2G and 2H). Interestingly, the mitochondria demonstrated clear structural changes in the midgut of QND-treated insects, but observations were plagued by low abundance of this organelle (Fig 2D, 2G and 2H). These effects include swollen and washed-out mitochondrial matrix, suggesting increased permeability transition of the inner membrane, a pre-requisite for mitophagy [29] (Fig 2G and 2H). Indeed, mitochondria were frequently observed within autophagosomes in posterior midguts of QND-treated insects, as shown in Fig 2H. Despite the massive changes in midgut architecture, the homogeneous and pronounced distribution of microvilli along luminal epithelial border (see "asterisks" on Fig 2C, 2D and 2E), and the preserved capacity to digest blood (S1E and S1F Fig), indicate that midgut integrity, and function were preserved upon QND treatment. Contrasting with the general organelle disappearance, extensive cytosolic electron-dense structures that resemble residual bodies (hemoxisomes, [30]) accumulate in the midgut cells of QND-treated insects (Fig 2D and 2E). Thereby, maintenance of low "free" heme levels in the midgut lumen through its crystallization into Hz preserves midgut cellular architecture.

### Impaired Hz formation causes systemic heme overload and redox imbalance, while activates a compensatory heme-scavenging mechanism

**Fig 3:**
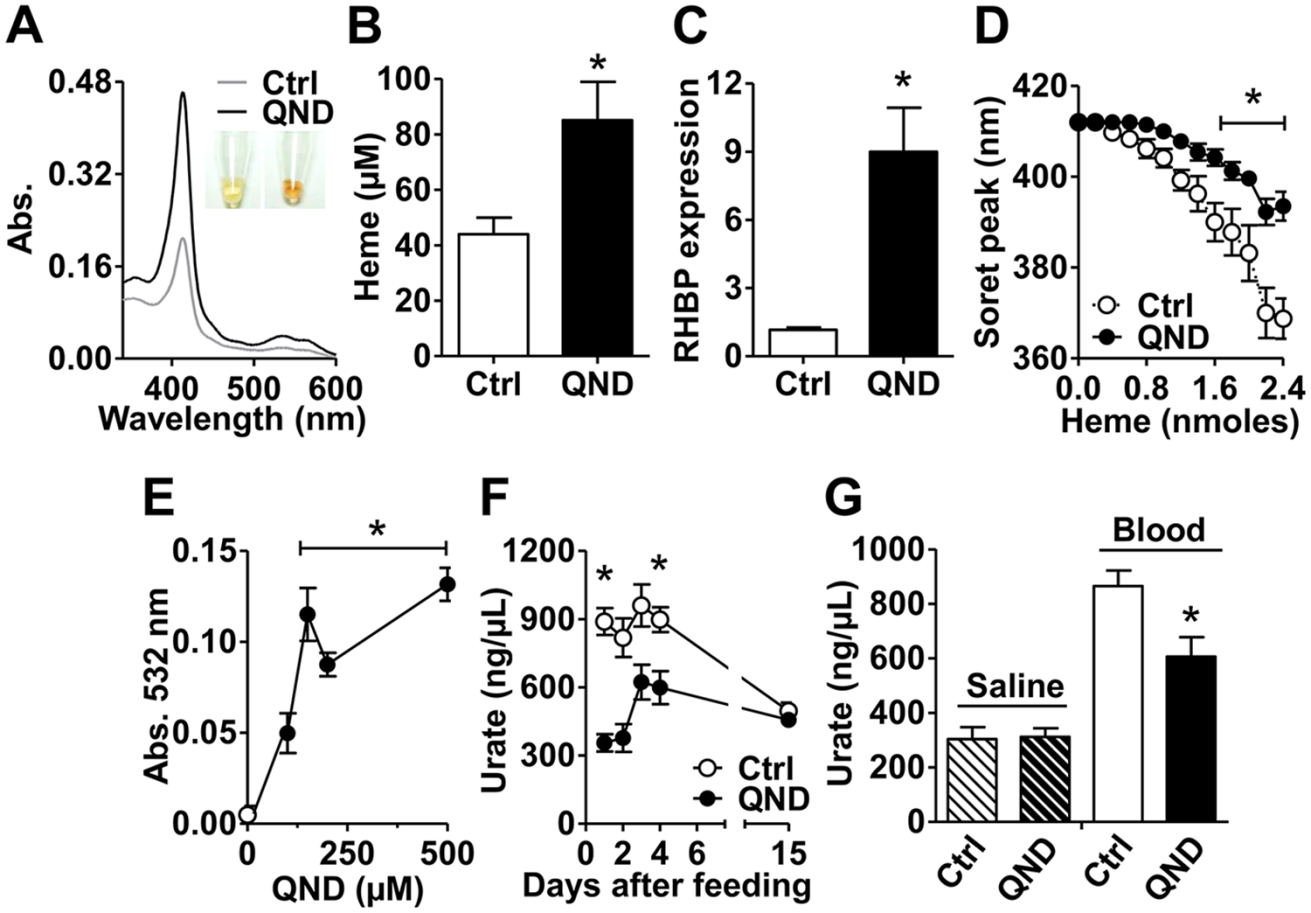
Limited Hz formation causes systemic heme overload, activation of compensatory heme detoxification mechanisms, and redox imbalance. Insects were fed with blood (Control, Ctrl), or blood supplemented with quinidine (QND) and analyzed four days (**A-E,G**) or along fifteen days (**F**) after feeding. All experiments were conducted using insects from IBqM colony. **(A)** Light absorption spectra of hemolymph. Insets show the dark reddish-color of hemolymph from control and QND treated insects. **(B)** Total heme concentrations in the hemolymph. Ctrl: n = 17; 100 μM QND: n = 16. Comparisons between groups were done by Student’s t test (*p<0.01). **(C)** Relative expression of *Rhodnius* heme-binding protein (RHBP) in fat bodies. Ctrl: n = 3; 100 μM QND: n = 3. Comparisons between groups were done by Student’s t test (*p<0.005 relative to Ctrl). **(D)** Heme buffering capacity of hemolymph from the insects. Ctrl: n≥4; 100 μM QND: n≥3. Comparisons between groups were done by two-way ANOVA and *a posteriori* Bonferroni’s tests (*p<0.05). **(E)** Lipid peroxide levels in the hemolymph. Ctrl: n = 4; QND: n≥6. Comparisons between groups were done by one-way ANOVA and *a posteriori* Tukey’s tests (*p<0.05 relative to Ctrl). **(F)** Urate levels in the hemolymph. Ctrl: n≥3; 100 μM QND: n≥3. Comparisons between groups were done by two-way ANOVA and *a posteriori* Bonferroni’s tests (*p<0.05 relative to Ctrl). **(G)** Urate levels in the hemolymph from insects fed with saline (Ctrl, n = 8), saline supplemented with 100 μM QND (n = 9), blood (Ctrl, n = 44), or blood supplemented with 100 μM QND (n = 46). Comparisons between groups were done by Mann Whitney´s test (*p<0.0005 relative to Blood Ctrl). Data in Figs 3B-3G were expressed as mean ± S.E.M.

To determine the consequences of impaired Hz formation at systemic levels, we evaluated in the next set of experiments hemolymphatic heme properties and redox homeostasis markers. The results showed that the hemolymph from QND-fed insects were reddish in color compared to controls (Fig 3A Inset), exhibiting higher levels of "free" and protein-bound heme, as measured by light absorption at 365 nm and 412 nm, respectively (S2C and S2D Fig). Direct quantification of total heme showed that inhibition of Hz formation in the midgut caused a remarkable increase in hemolymph (Fig 3B) and heart (S3E Fig) heme levels. The high hemolymphatic levels of heme after QND treatment increased the expression of *Rhodnius* Heme Binding Protein (RHBP) in about 7.5 times in fat bodies (Fig 3C and S2F Fig), which is consistent with the antioxidant role of this protein by preventing the pro-oxidant effect of heme [31,32]. Higher RHBP levels conferred improved hemolymph buffering capacity against the "free" heme, as revealed by higher resistance of hemolymph to undergo blue shift the Soret peak after heme titration (Fig 3D). Moreover, heme overload did not significantly affect biliverdin production in the heart (S2G Fig), suggesting that *Rhodnius* heme oxygenase (HO) activity [33] might be saturated, thus not significantly contributing to heme detoxification. Despite the improved heme buffering capacity by RHBP (Fig 3D), we observed systemic redox imbalance, as measured by higher lipid peroxide levels (Fig 3E), and reduced concentrations of the main low molecular weight antioxidant urate in the hemolymph of adults (Fig 3F and S2H Fig) and of young nymphs (S2I and S2J Fig) from both colonies upon QND treatment. Reduction in urate levels by QND is directly related to defective heme crystallization, and not related to inhibition of urate synthesis, as this effect was only observed in blood fed insects (Fig 3G). Supporting this proposal, reductions in urate levels were more pronounced exactly at the times of highest Hz production rates (Fig 1B and S2A Fig). The data presented here indicate that systemic heme overload upon QND treatment causes redox imbalance, while activating a compensatory heme-scavenging mechanism (RHBP) in the hemolymph.

### Oogenesis and *T*. *cruzi* infection are reduced upon impairment of heme crystallization by redox imbalance

**Fig 4:**
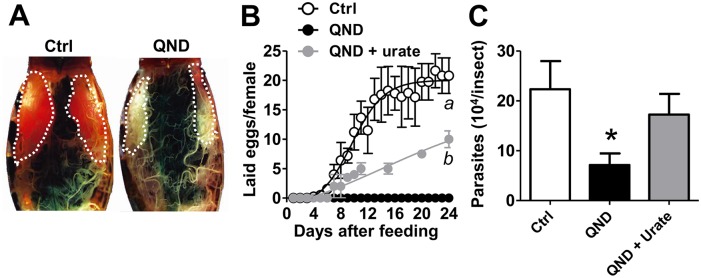
Oogenesis and *Trypanosoma cruzi* infection depend on heme crystallization in the midgut. Insects were fed with blood (Ctrl) or blood supplemented with 100 μM QND or blood supplemented with 100 μM QND and 1 mM urate (QND+urate). **(A)** Stereoscope images of adult female abdomens from IBqM colony four days after feeding. Ovaries are depicted as dashed white lines. **(B)** The average number of eggs laid per females from Fiocruz colony insects fed with blood was determined along 24 days after blood meal. Ctrl: n≥3; 100 μM QND: n≥3; QND+urate: n≥3. Comparisons between groups were done by two-way ANOVA and a *posteriori* Bonferroni’s tests (^a^p<0.0001 relative to QND and QND+urate, and ^b^p<0.0001 relative to QND). **(C)** Total *T*. *cruzi* counts in the digestive tract of infected adult females from Fiocruz colony 15 days after blood meal. Ctrl: n = 18; 100 μM QND: n = 18; QND + urate: n = 8). Comparisons between groups were done by one-way ANOVA, (*p = 0.007 relative to Ctrl), with a *posteriori* Bonferroni’s tests. Data in Fig 4B were expressed as mean ± S.E.M., and in Fig 4C as scattered plot with gray lines representing medians.

As shown above, impairment of Hz formation *in vivo* promotes redox imbalance (Figs 1C, 1D, 3E, 3F and 3G, S2H, S2I and S2J Fig), which is a key mechanism to control fertility in different models [11,34,35]. Then, we determined the potential consequences of QND treatment on *R*. *prolixus* reproduction, by assessing the number of eggs produced and laid per female. Unlike control insects, blockage of heme crystallization by QND caused a remarkable effect on ovary development and egg production, with no apparent pinkish-colored eggs observed four days after a blood meal (Fig 4A). This remarkable effect on oogenesis was sustained along the full blood digestion and reproductive cycle, in both insect colonies (Fig 4B and S3 Fig). Interestingly, the reduced egg production caused by QND involves redox imbalance, as supplementation in the diet with the major antioxidant urate restored oogenesis in QND-treated insects (Fig 4B). This indicates that heme crystallization in the midgut determines oogenesis in triatomines by systemically regulating redox balance.

Interestingly, QND strongly reduced *T*. *cruzi* parasite loads in *R*. *prolixus* digestive tract (by ~ 75%) 15 days after blood meal (Fig 4C). Similar effects were also observed at earlier (S4A Fig), and later (S4B Fig) times of blood digestion. To demonstrate whether parasites were affected by the pharmacological inhibition of Hz formation, we assessed the effects of QND and heme on *in vitro* epimastigotes and trypomastigotes cultures of *T*. *cruzi*. Although incubation of epimastigotes with 30 μM heme promoted parasite proliferation by ~ 55%, co-incubation with 50–100 μM QND completely blunted this effect (S4C Fig). On the other hand, heme exerted a strong cytotoxic effect to trypomastigotes, reducing parasite counts by ~ 50%, which was not affected by the presence of 50–100 μM QND (S4D Fig). These data indicate that the effect on the parasites is not due to the QND administration *per se*. On the other hand, dietary supplementation with urate restored parasite count *in vivo* to levels similar to the control group (Fig 4C). In summary, these results indicate that heme crystallization in triatomine midgut represents a key mechanism to allow *T*. *cruzi* proliferation and survival, by keeping heme at strict concentrations, enough to boost epimastigote growth, but without leading to an excessive redox imbalance which culminates in parasite death.

## Discussion

**Fig 5:**
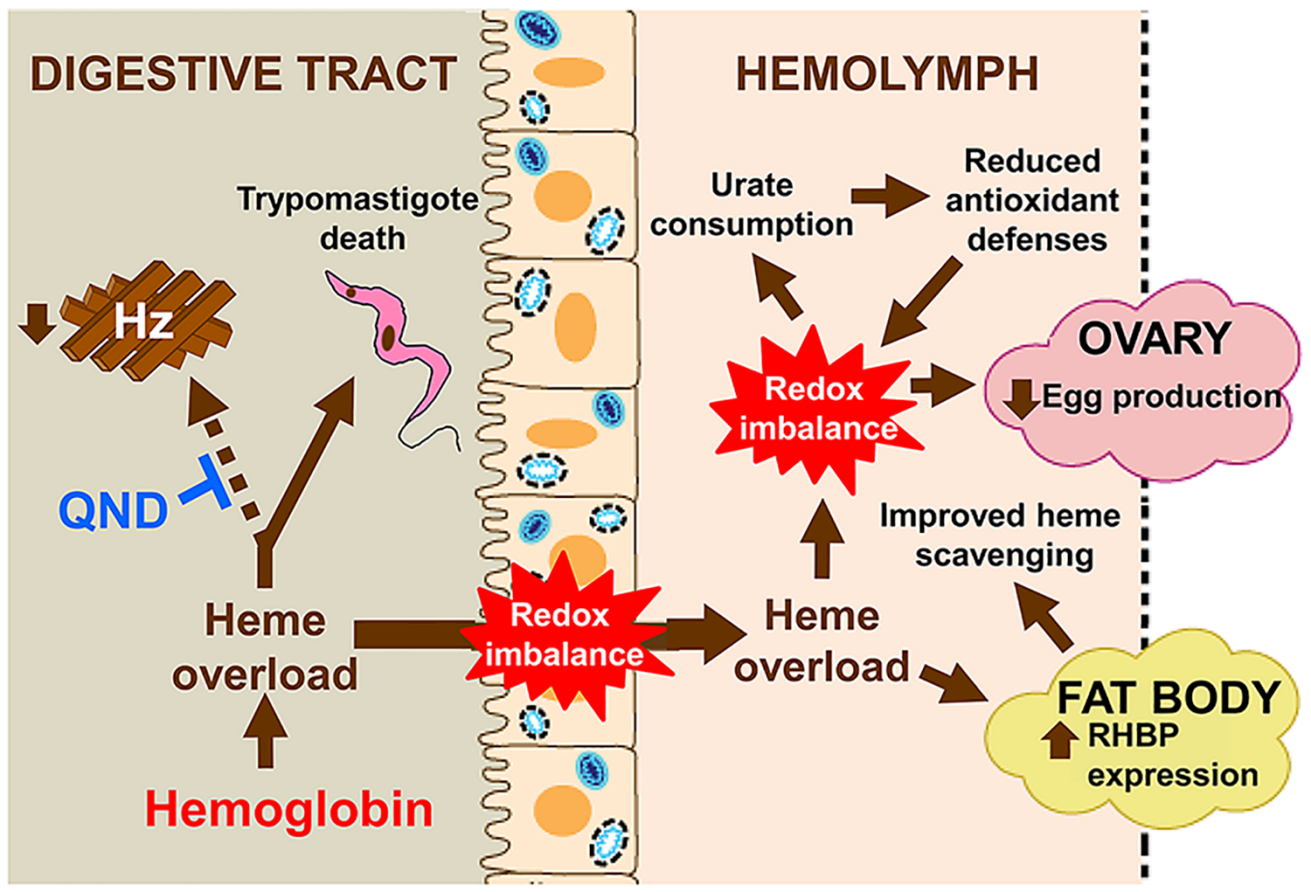
Schematic model of physiological consequences of blocked Hz formation in triatomine midgut. In the presence of QND, heme derived from blood meal forms stable complexes with this drug, impairing Hz formation in the midgut lumen. Non-crystallized heme levels build up in the midgut causing cytotoxic effects to *T*. *cruzi* trypomastigotes. Excessive heme is transported to hemolymph through the midgut cells by hemoxisomes/residual bodies, causing redox imbalance and autophagy in the midgut. Heme accumulates in the hemolymph, increasing RHBP production, as a compensatory defense against "free" heme. However, this mechanism is overwhelmed, as the levels of urate drop. Redox imbalance has a direct effect on oogenesis, reducing egg production.

We describe here an unprecedented function for Hz production in the triatomine insect *R*. *prolixus* that efficiently reduces redox heme reactivity allowing insect fertility and *T*. *cruzi* life-cycle. To our knowledge, this is the first comprehensive description of disrupted heme crystallization in a CD vector and identifies the unique role of Hz as a key redox-protective mechanism for both triatomine vector and for *T*. *cruzi* parasites. A summary of the effects resulted from impaired Hz formation in *R*. *prolixus* is schematically depicted in Fig 5.

As obligatory blood feeders, triatomine insects ingest copious amounts of blood to meet their energy demands, and hemoglobin is degraded into peptides and amino acids, releasing ~ 10 mM heme in the midgut lumen [3,36]. This massive heme release poses a major threat to triatomines [10,11,13,31], owed the cytotoxic properties of heme [9,23,25,27,37]. The primary and major defensive mechanism against heme toxicity in triatomines consists on its crystallization into Hz. When this process was inhibited by QND treatment, “free” heme accumulates in the midgut lumen, a proposal that is experimentally sustained by the following observations: i) blood intake (S1C and S1D Fig), and digestion (S1E and S1F Fig) were not affected by QND, implying that heme supply for crystallization was not limited; ii) Hz formation was significantly affected by QND both in adult (Fig 1A and 1B, and S2A Fig), and nymph stages (S2B Fig); iii) hemoxisomes/residual bodies increased in density with QND treatment (Fig 2A and 2B); and iv) hemolymph (Fig 3A and 3B, S2C and S2D Fig), and heart (S2E Fig) were overloaded with heme upon QND treatment. From the parasite side, although excessive “free” heme levels in the digestive tract are not harmful to *T*. *cruzi* epimastigote forms [6,7], it exerts powerful cytotoxic effects to metacyclic trypomastigotes [7]. In fact, the proliferative effects of heme to epimastigote forms depend on oxidants generated by parasite mitochondria [6–8], while trypomastigotes require reduced environments to allow differentiation [7]. In line with these observations, inhibition of Hz formation reduced total parasite counts in *R*. *prolixus* digestive tract (Fig 4C, S4A and S4B Fig). Also, QND inhibited the proliferative effects of heme on epimastigotes (S4C Fig), while did not potentiate the cytotoxic effect of heme on trypomastigotes (S4D Fig). Therefore, we concluded that reductions in *T*. *cruzi* counts in triatomine midgut were most likely a consequence of the cytotoxic effect of excessive “free” heme on trypomastigotes rather than in epimastigotes. Despite the fact that QND promoted clear effects on triatomine heme/redox homeostasis, we cannot rule out the potential off-target effects of QND on *T*. *cruzi* parasite forms. In this sense, one might consider that reduced *T*. *cruzi* counts upon QND treatment results from lower urate levels, independently of its redox properties [7,38]. The differential susceptibility to excessive heme/oxidants may result from specific adaptations developed by *T*. *cruzi* forms to cope with environmental challenges that parasites face during their development [6–8,39]. Conceivably, Hz formation would provide a suitable and unique environment for trypanosomes to develop through the triatomine digestive tract, by maintaining “free” heme levels high enough to induce epimastigotes proliferation, without compromising metacyclogenesis and trypomastigotes survival.

Although midgut represents the first line of defense against multiple stressors coming from the diet, its cells are not completely immune to handle “free” heme overload. In the process of transporting “free” heme from the midgut lumen to the hemolymph, midgut cells also facilitate the production and propagation of oxidants (Fig 1C and 1D), causing redox imbalance. Supporting these observations, inhibition of Hz production in malaria parasites was also associated to redox imbalance [25,40]. A second phenotype observed is the remarkable architectural change undergone by midgut cells upon QND treatment (Fig 2). Extensive organelle disappearance (Fig 2D), cytosolic vacuolization (Fig 2B and 2D), enrichment of intracellular lipid droplets (Fig 2B and 2E), and increase in structures with concentric membranes, similar to autophagosomes (Fig 2H) were all evident in QND treated insects. Moreover, mitochondrial disappearance was also a hallmark in QND treated insects, and the few detected exhibited remarkable structural changes, including clear matrix spaces (Fig 2G), with fewer cristae compared to controls. Mitochondrial structural changes are indicative of reduced functionality, which might precede the extensive organelle elimination by autophagy. Similar ultrastructural observations were reported for *Plasmodium* and *Schistosoma* parasites under aminoquinoline treatment, including mitochondrial swelling, cytoskeletal disorganization, and autophagy [24,41,42]. Indeed, higher oxidant levels resulting from accumulation of porphyrins [43], or iron [44] in different models point out to autophagy as a potential protective mechanism for cell quality control during iron/heme overload. Interestingly, given that proteases involved in hemoglobin degradation are produced by midgut cells [36], and that blood digestion was not affected by QND treatment (S1E and S1F Fig), the function of midgut cells was preserved despite the massive organelle removal, structural changes, and redox imbalance.

Inhibition of Hz formation increased the flux of heme through the midgut leading to heme overload in the hemolymph (Fig 3B, S2C and S2D Fig) and the heart (S2E Fig). As a consequence, systemic redox homeostasis shifted towards oxidation, as measured by higher lipid peroxide levels (Fig 3E), and lower levels of the antioxidant urate in the hemolymph of QND treated insects (Fig 3F and 3G, S2H, S2I and S2J Fig). Despite the altered heme homeostasis, biliverdin levels in the heart were not significantly changed by limited Hz production (S2G Fig), suggesting that HO activity was not affected by QND. In line with these observations, the expression of the main heme scavenging protein RHBP significantly increased upon inhibition of Hz formation (Fig 3C and S2F Fig), improving the buffering capacity of “free” heme in the hemolymph (Fig 3D). Hemolymph light absorption at 412 nm, which reflects heme binding to RHBP [31,32], significantly increased under QND treatment (Fig 3A, S2C and S2D Fig), indicating that most of “free” heme was scavenged by apo-RHBP. Previous reports demonstrated that RHBP expression peaks ~ 2–4 days after blood meal, suggesting that heme regulates RHBP expression [45], a proposal that is experimentally supported by the present work (Fig 3B, 3C and 3D, S2C, S2D, S2E and S2F Fig). Despite the compensatory increase in heme buffering capacity provided by higher apo-RHBP levels under blockage of Hz formation (Fig 3D), “free” heme accumulates in the hemolymph, as revealed by the higher absorption at 365 nm (S2D Fig) [46]. Thus, higher “free” heme titers in QND treated insects overwhelms the buffering capacity of RHBP, allowing heme to exert its pro-oxidant effects (Fig 3E, 3F and 3G). Urate is a key antioxidant in triatomines [47], and it is synthesized in the fat body upon heme signaling involving PKC activity [48]. In spite of heme overload, urate levels reduced upon QND treatment (Fig 3F and 3G, S2H, S2I and S2J Fig), which might indicate urate overconsumption to counteract higher oxidant levels propagated by heme and to minimize redox damage in the hemolymph. Therefore, inhibition of Hz formation is not lethal to triatomines, as opposed to malaria parasites, but rather shifts the heme detoxification mechanisms from heme crystallization towards its scavenging by a chelating protein (RHBP).

In this study, we have observed a delay and a decrease of oogenesis and egg deposition as a consequence of impaired Hz formation (Fig 4A and 4B and S3 Fig). This effect was partially reversed by dietary urate supplementation, suggesting that redox imbalance generated by heme overload represents an important mechanism that mediates reduced oogenesis (Fig 4B). Due to the partial restoring effect of urate on egg production, we cannot completely exclude the possibility that QND might exert direct effects on oogenesis by an unknown redox-independent mechanism. In this regard, similar tradeoffs between redox homeostasis and reproduction were reported for distinct models, including blood feeding insect vectors [11,34]. Also, impairment in heme scavenging through the knockdown of RHBP in triatomines, caused lipid peroxidation, reducing mitochondrial function, and energy supply, with direct effects on embryogenesis, but not oogenesis [11]. The emerging picture is that RHBP serves not only as a preventive antioxidant, by sequestering "free" heme into an inert complexed form, but also as a mechanism for heme delivery to maintain a proper energy supply during embryogenesis. Thus, despite the fact that impairment of Hz formation and RHBP knockdown converge towards "free" heme overload in the hemolymph, the biological outputs were contrasting: while QND treatment impaired oogenesis (Fig 4B and S3 Fig), RHBP silencing reduced embryogenesis [11]. These observations can be reconciled if we consider that the redox insult generated upon interference of Hz formation is much stronger than observed after RHBP knockdown, and, potentially, that embryogenesis would be a more sensitive process to oxidants than oogenesis in triatomines.

Living organisms have evolved distinct mechanisms to avoid the potential cytotoxic effects of "free" heme overload [49]. In systems that are not physiologically adapted to high heme levels, such as mammals, heme is essentially detoxified through its enzymatic degradation by HO activity, or scavenged by heme binding proteins [49–51]. However, depending on the magnitude of heme exposure, these protective mechanisms might be saturated and result in tissue damage. Conversely, natural exposure to high levels of heme, as in the case of hematophagous organisms, posed a selective pressure to evolve efficient and unique mechanisms to cope with heme overload [3,12]. Given the central importance of heme homeostasis for both triatomine insect vector [3,12], (Figs 1–5), and *T*. *cruzi* parasites [4–9], (Fig 4 and S4 Fig), interference on Hz formation disrupts vector-parasite interaction, with detrimental effects on CD transmission. In conclusion, we present here, to our knowledge, the first comprehensive description of physiological, biochemical, cellular, and molecular consequences of disrupted heme crystallization in a CD vector. Collectively, our data highlight the dual role of Hz as a key protective mechanism, with striking beneficial outputs for both triatomine vector and for *T*. *cruzi*. The possibility to target such a central redox process for both vector and parasite represents a major step towards the development of innovative and rational strategies for effective CD control.

## Materials and methods

### Ethics statement

*R*. *prolixus* were fed and raised according to the Ethical Principles in Animal Experimentation approved by the Ethics Committee in Animal Experimentation (CEUA) at Federal University of Rio de Janeiro, CEUA/UFRJ), and at Fundação Oswaldo Cruz (CEUA/FIOCRUZ), under the approved protocols #IBQM050, and P-54/10-4/LW12/11, respectively. Experiments with citrated human blood were carried out using insects from Fiocruz colony and were fed by using an artificial apparatus according to the Ethical Principles in Animal Experimentation approved by the Ethics Committee in Animal Experimentation (CEUA/FIOCRUZ) under the approved protocol L-0061/08. All blood donors provided informed written consent. All protocols are from CONCEA/MCT (http://www.mctic.gov.br/mctic/opencms/institucional/concea/index.html), which is associated with the American Association for Animal Science (AAAS), the Federation of European Laboratory Animal Science Associations (FELASA), the International Council for Animal Science (ICLAS) and the Association for Assessment and Accreditation of Laboratory Animal Care International (AAALAC). Technicians dedicated to the animal facilities at the Institute of Medical Biochemistry, and at Fundação Oswaldo Cruz, carried out all aspects related to rabbit husbandry under strict guidelines to ensure careful and consistent handling of the animals.

### Insects

*Rhodnius prolixus* insects used throughout this work were from two different well-established colonies: i) Institute of Medical Biochemistry (IBqM) at Federal University of Rio de Janeiro (UFRJ, Brazil) and ii) Laboratory of Insect Physiology at Oswaldo Cruz Institute (Fiocruz, Rio de Janeiro, Brazil). The reason to use two colonies aimed the exclusion of potential external (colony handling/maintenance, blood source), and internal (microbiota, genetic background) interference factors that might eventually bias the results. The trends observed throughout the data from both colonies were strikingly similar, only differing in the magnitude of the responses. All insects were kept at 28°C and 60–80% relative humidity, with a photoperiod of 12 h of light/12 h of dark, as previously described [52]. Insects from IBqM colony were maintained by feeding directly on rabbit ears, while individuals from Fiocruz colony were maintained by feeding artificially with defibrinated rabbit blood using an artificial feeder [53].

### In vitro cultures of *T*. *cruzi*

*T*. *cruzi* Dm28c strain (CT-IOC-010) was provided by the Trypanosomatid Collection of the Oswaldo Cruz Institute, Fiocruz, Brazil. Epimastigote parasite forms were grown at 28°C for 7 days in brain-heart infusion medium (BHI, BD Bacto, BD Biosciences, USA) + 10% fetal bovine serum (Vitrocell, Brazil), and were further incubated for 5 days with 30 μM heme, or 30 μM heme + 50–100 μM QND in 96-well plates. Metacyclogenesis was performed as described elsewhere [54], and resulting trypomastigotes were incubated for 2 days with 30 μM heme, or 30 μM heme + 50–100 μM QND in 96-well plates. Parasites in the culture supernatant were collected, and survival was determined by cell counting of viable cells in a Neubauer chamber.

### Dietary QND supplementation

For both insect colonies, treatments were performed in adult mated *R*. *prolixus* females from their second cycle after the last ecdysis, while young nymph stages were from the fifth cycle before the last ecdysis. To inhibit Hz formation *in vivo*, all insects were artificially fed with rabbit blood supplemented with either 0.6% (v/v) ethanol (control) or different quinidine concentrations (0–500 μM QND) previously prepared from 30% ethanol or 5 mM QND in 30% ethanol stocks and maintained for up to 24 days after blood meal. To control the volume of blood ingested by each group, the insects were weighted before and after blood feeding, and no significant changes were reported in the volume of engorged blood (S1C and S1D Fig).

### *In vivo* effects of QND on *T*. *cruzi*

For the *T*. *cruzi* infection experiments, insects from Fiocruz colony at their first cycle after the last ecdysis were fed with heat-inactivated citrated human blood supplemented with 10^8^ epimastigotes/mL (Dm28c strain) as described elsewhere [55]. After 28 days, insects were artificially fed with rabbit blood (control), or blood supplemented with 100 μM QND. Viable parasite counts were determined in the whole midgut and monitored 10, 15 and 30 days upon feeding with QND by direct counting of parasites in a Neubauer chamber.

### Quantification of hemoglobin in anterior midgut

Four days after feeding, anterior midguts were homogenized in PBS, and general protease inhibitor cocktail (Sigma, catalog number P2714) and stocked at -80°C until the measurement. Hemoglobin in anterior midgut was quantified by using a commercially available kit (Bioclin, Brazil). Reagent solution was diluted 1:100 in water and 2.5 mL were added to 10 μL of sample. Experiments were performed in 96-well plates and the absorbance at 540 nm was determined after 5 min at room temperature in a Molecular Devices Spectra Max M5spectrophotometer and hemoglobin concentration was calculated using hemoglobin (Bioclin, Brazil) as standard.

### Hemozoin extraction

Posterior midguts were dissected under PBS and the luminal contents were collected, homogenized and centrifuged at 11,000×g for 10 min. The pellet was re-suspended in 0.1 M sodium bicarbonate buffer, pH 9.1 and 2.5% SDS. Samples were centrifuged at 11,000×g for 10 min and the pellet was washed five times with the same buffer, followed by two washes with deionized water. Hz quantification was carried out by adding 1 mL of 0.1 M NaOH to the pellet, vortexing the samples for 30 min, followed by determination of heme content at 400 nm in a GBC-UV/Vis-920 spectrophotometer, using a standard curve made with hemin (Frontier Scientific, USA) dissolved in 0.1 M NaOH.

### Quantification of midgut oxidant levels

To assess oxidant levels, the wings, legs and dorsal plaques were dissected from the insects, and the hemocoel was filled with a 50 μM solution of oxidant-sensitive fluorophore dihydroethidium (hydroethidine, DHE) (Invitrogen, USA) diluted in L15 culture medium (Gibco, USA) containing 5% (v/v) fetal bovine serum. The samples were incubated in the dark at 28^°^C. After 20 min of incubation, the midguts were washed with 0.15 M NaCl, and immediately transferred to a glass slide for fluorescence microscopy analysis, as previously described [13]. Each condition was registered in at least three different areas for fluorescence, and differential interference contrast (DIC). Quantitative evaluation of fluorescence levels was performed by acquiring images under identical conditions using an objective of 20 x and 100 ms exposure time in each experiment. The images were acquired in a Zeiss Observer Z1 fluorescence microscope with a Zeiss Axio Cam MrM, and data were analyzed using AxioVision version 4.8 software. The #15 filter set (excitation BP 546/12 nm; beam splitter FT 580 nm; emission LP 590 nm) was used.

### Midgut histology and ultrastructure

Posterior midguts of adult females were collected and fixed in 2.5% of glutaraldehylde in 0.1 M sodium cacodylate buffer pH 7.2 for at least 24 h at 4°C, followed by a post-fixation in 1% osmium tetroxide, 0.8% potassium ferricyanide and 2.5 mM calcium chloride in the same buffer for 1 h at room temperature. The dehydration steps were performed by incubations of 15 min for each acetone concentration (30%, 50%, 70%, 90% and 100%). Then the samples were gradually embedded in epoxy polybed resin (Polybed 812, Polysciences, Germany). Infiltration was performed by incubating samples with 1:3, 1:2, 2:3 of epoxy polybed resin:acetone for 24 h to each step. After that, samples were infiltrated with epoxy polybed resin 100%, incubated at room temperature for 4 h and then incubated at 60°C for 4 days to complete polymerization. Then, semi-thin sections (0.5 μm) were obtained, stained with toluidine blue and observed in a Zeiss Axioplan bright field microscope for histological analysis. Alternatively, for ultrastructural analysis, ultrathin sections were obtained with an ultramicrometer Ultracuts (Leica) collected in copper grids, stained in uranyl acetate for 20 min and lead citrate for 2 min, and sections were observed in a JEOL JEM1011 transmission electron microscope at Oswaldo Cruz Institute electron microscopy platform.

### Biochemical analyses of hemolymph

Hemolymph was collected before blood meal and 1, 2, 3, 4, 7 and 15 days after feeding, in tubes containing a few crystals of phenylthiourea, by cutting a leg, and applying a gentle pressure to the insect abdomen. Qualitative analyses of heme levels were assessed 4 days after feeding by diluting a hemolymph aliquot (15 μL) in 485 μL PBS pH 7.4, and the light absorption spectra was analyzed between 300 nm and 800 nm in a Shimadzu UV-2550 spectrophotometer. Absolute quantification of total heme was determined by the alkaline pyridine-hemochrome method, using the reduced minus oxidized spectra as described elsewhere [56]. Quantification of "free" and RHBP complexed heme were determined in hemolymph specifically at 365 nm and 412 nm, respectively [32,46]. Heme binding to *Rhodnius* heme binding protein (RHBP) was measured by progressively adding 2 μL of 0.1 mM heme solution (Frontier Scientific, USA) as previously described [32] using a Shimadzu UV-2550 spectrophotometer. Lipid peroxidation was assessed by quantifying the levels of thiobarbituric acid reactive substances assay (TBARS) at 532 nm in a Molecular Devices Spectra Max M5 spectrophotometer as described elsewhere [10]. To determine urate levels, 3 μL of hemolymph was diluted 1:5 in water and subsequently urate concentration was enzymatically determined using a commercially available kit (Doles, Brazil) following the manufacturer’s instructions and using urate (Doles, Brazil) as standard as previously described [47].

### Quantification of heme metabolites in heart

Ten heart samples per group were collected four days after feeding, homogenized with 200 μL 5% acetonitrile and 0.05% TFA as solvent, pH 2.0 (1:2 v/v), centrifuged for 15 min at 12,000×g and the supernatant was applied onto a Shimadzu CLC-ODS C18 column (15 mm × 22 cm) equilibrated with the same solvent, using a flow rate of 0.4 mL/min. After 10 min, an acetonitrile linear gradient (5–80%) was applied for 10 min, followed by 20 min of 80% acetonitrile with 0.05% TFA, pH 2.0, and heme and biliverdin peaks were identified as previously described [33]. The experiments were performed at room temperature.

### Tissue isolation and RNA extraction

Fat bodies from fed females were dissected 4 days after feeding. Total RNA was extracted from 1 fat body per tube containing 1 mL of TRIzol reagent (Invitrogen, USA), according to the manufacturer’s instructions and at least 3 insects were used per experiment. RNA concentrations were determined spectrophotometrically at 260 nm on a Nanodrop 1000 spectrophotometer v.3.7 (Thermo Fisher Scientific, USA). Following treatment with 1 U RNase-free DNase I (Fermentas International Inc., USA) for 5 min at 37°C, 1 μg of RNA was used for cDNA synthesis with a high capacity cDNA reverse transcription kit (Applied Biosystems, USA) and random hexamers according to the manufacturer’s instructions.

### Quantitative RT-PCR

Quantitative PCR was performed in a 7500 real time PCR system (Applied Biosystems, USA) using SYBR Green PCR Master Mix (Applied Biosystems, USA) under the following conditions: one cycle for 10 min at 95 °C, followed by fifty cycles of 15 s at 95 °C and 45 s at 60 °C. PCR amplification was performed using the following primers: qRHBPF (5′-TCCTTCACACTCTCCGCAAC-3′) (forward) and qRHBPR (5′-GTACGCTTGGTACGCCACTT-3′) (reverse) [11]. Three independent biological replicates were conducted, and all PCRs were performed in triplicate. *R*. *prolixus* actin gene (accession number EU2337941) expression was used as an internal control for normalization. Primers used for actin PCR amplification were RpActRT F (5′-CCATGTACCCAGGTATTGCT-3′) (forward) and RpActRT R (5′-ATCTGTTGGAAGGTGGACAG-3′) (reverse) [11]. ΔΔCt values were calculated from Ct (cycle threshold) values obtained on quantitative RT-PCR and were used to calculate relative expression and perform statistical analysis [57]. Differences were considered significant when p<0.05. The relative expression values based on 2-ΔΔCt were used only for graphic construction.

### Egg production

Oogenesis and egg laying were determined along 24 days after blood feeding, by direct counting laid eggs.

## Supporting information

S1 Fig Triatomine survival, blood ingestion and digestion were not affected by QND treatment. Adult females from different colonies were fed with blood (Control, white circles) or blood supplemented with 100 μM quinidine (QND, black circles). **(A and B)** Survival of triatomines from IBqM (**A**, n = 6), or Fiocruz colonies (**B**, n = 5) were determined along 24 days upon blood meal. **(C and D)** The average volume (μL) of blood engorged per insect was assessed in insects fed with blood (Control, white bars), or blood supplemented with 100 μM quinidine (QND, black bars), from IBqM (**C**, n≥41) or Fiocruz (**D**, n≥21) colonies four days after feeding. Data are expressed as mean ± S.E.M. **(E and F)** Blood digestion was determined by hemoglobin quantification in the anterior midgut from insects fed with blood (Control, white bars), or blood supplemented with 100 μM quinidine (QND, black bars), from IBqM (**E**, n≥12) or Fiocruz (**F**, n = 2) colonies four days after feeding. Data are expressed as mean ± S.E.M.
(PPTX)

S2 Fig Quinidine inhibits Hz formation in triatomine adults and nymphs, promoting heme overload and redox imbalance in the hemolymph. **(A)** Adult females from Fiocruz colony were fed with blood (Control, white circles, n≥3), or blood supplemented with 100 μM quinidine (QND, black circles, n≥3), and Hz content in posterior midgut was determined along 15 days after blood meal. Comparisons between groups were done by two-way ANOVA and *a posteriori* Bonferroni’s tests, with *p<0.01 relative to control. **(B)** Fifth-instar nymphs from IBqM colony were fed with blood (Control, white bar, n = 6) or blood supplemented with 100 μM quinidine (QND, black bar, n = 6), and Hz content in posterior midgut was determined four days after blood meal. Comparisons between groups were done by Mann Whitney’s test, with *p<0.03 relative to control. Data are expressed as mean ± S.E.M. **(C and D)** Light absorption of hemolymph from adult insects fed with blood (control, white bars, n = 11) or blood supplemented with 100 μM quinidine (QND, black bars, n = 13), from IBqM colony spectrophotometrically determined at 365 nm and 412 nm. Comparisons between groups were done by Student’s *t* test, with *p<0.05 or **p<0.001 relative to control. Data were expressed as mean ± S.E.M. **(E)** Total heme levels in hearts from adult insects fed with blood (control, white bars, n = 3) or blood supplemented with 100 μM quinidine (QND, black bars, n = 3), from Fiocruz colony were determined by HPLC four days after blood meal. **(F)** Relative expression of *Rhodnius* heme-binding protein (RHBP) in fat bodies of insects fed with blood (control, white bars, n = 2) or blood supplemented with 100 μM quinidine (QND, black bars, n = 2), from Fiocruz colony was assessed by qPCR four days after blood meal. **(G)** Total biliverdin content in hearts from adult insects fed with blood (control, white bars, n = 3) or blood supplemented with 100 μM quinidine (QND, black bars, n = 3), from Fiocruz colony were determined by HPLC four days after blood meal. **(H)** Urate levels in the hemolymph from adult insects fed with blood (control, white circles, n≥3) or blood supplemented with 100 μM quinidine (QND, black circles, n≥3), from Fiocruz colony were assessed spectrophotometrically along 15 days after blood meal. Comparisons between groups were done by two-way ANOVA and *a posteriori* Bonferroni’s tests, with *p<0.05 relative to control. **(I)** Urate levels in the hemolymph from fifth-instar nymphs fed with blood (control, white bar, n = 6) or blood supplemented with 100 μM quinidine (QND, black bar, n = 6), from IBqM colony were assessed spectrophotometrically four days after blood meal. Comparisons between groups were done by Mann Whitney´s test, with *p<0.005 relative to control. **(J)** Urate levels in the hemolymph from fifth-instar nymphs fed with blood (control, white bar, n = 4) or blood supplemented with 100 μM quinidine (QND, black bar, n = 6), from Fiocruz colony were assessed spectrophotometrically four days after blood meal. Comparisons between groups were done by Mann Whitney´s test, with *p<0.01 relative to control. Data were expressed as mean ± S.E.M.
(PPTX)

S3 Fig Blockage of Hz formation impairs oogenesis. The average number of eggs laid per females from IBqM colony insects fed with blood (control, white circles, n = 13), blood supplemented with 100 μM quinidine (QND, black circles, n = 15) was determined along 24 days upon blood meal. Comparisons between groups were done by two-way ANOVA and *a posteriori* Bonferroni’s tests, with ^a^p<0.0001 relative to control. Data were expressed as mean ± S.E.M.
(PPTX)

S4 Fig Impaired Hz formation reduces *Trypanosoma cruzi* infection and exerts cytotoxic effects on trypomastigote forms. **(A)** *T*. *cruzi* infected adult females from Fiocruz colony were fed with blood (control, white circles, n = 8), blood supplemented with 100 μM quinidine (QND, black circles, n = 8), and total parasite counts in the midgut were carried out after 5 days after blood meal using a Neubauer chamber. The red lines represent medians. **(B)** *T*. *cruzi* infected adult females from Fiocruz colony were fed with blood (control, white circles, n = 21), blood supplemented with 100 μM quinidine (QND, black circles, n = 15), and total parasite counts in the midgut were carried out after 30 days after blood meal using a Neubauer chamber. Comparisons between groups were done by Mann Whitney´s test, with *p<0.005 relative to control. The gray lines represent medians. **(C)** Total *T*. *cruzi* epimastigote counts after incubation for 5 days with 30 μM heme (white bar, n = 6) or 30 μM heme plus 50–100 μM quinidine (black bars, n = 6) was determined by cell counting in a Neubauer chamber. Data are expressed as mean ± S.E.M. Comparisons between groups were done by Kruskal-Wallis, and *a posteriori* Dunn´s tests, with *p<0.005 relative to control. **(D)** Total *T*. *cruzi* trypomastigote counts after incubation for 2 days with 30 μM heme (white bar, n = 4) or 30 μM heme plus 50–100 μM quinidine (black bars, n = 4) was determined by cell counting in a Neubauer chamber. Data are expressed as mean ± S.E.M. Comparisons between groups were done by Kruskal-Wallis, and *a posteriori* Dunn´s tests, with *p<0.005 relative to control.
(PPTX)
