# Supplementary figures and images for: Heme crystallization in a Chagas disease vector acts as a redox-protective mechanism to allow insect reproduction and parasite infection
Source: PLoS Negl Trop Dis. 2018 Jul 23;12(7):e0006661. doi: 10.1371/journal.pntd.0006661 (PMC6084092; doi:10.1371/journal.pntd.0006661)

## Slide 1
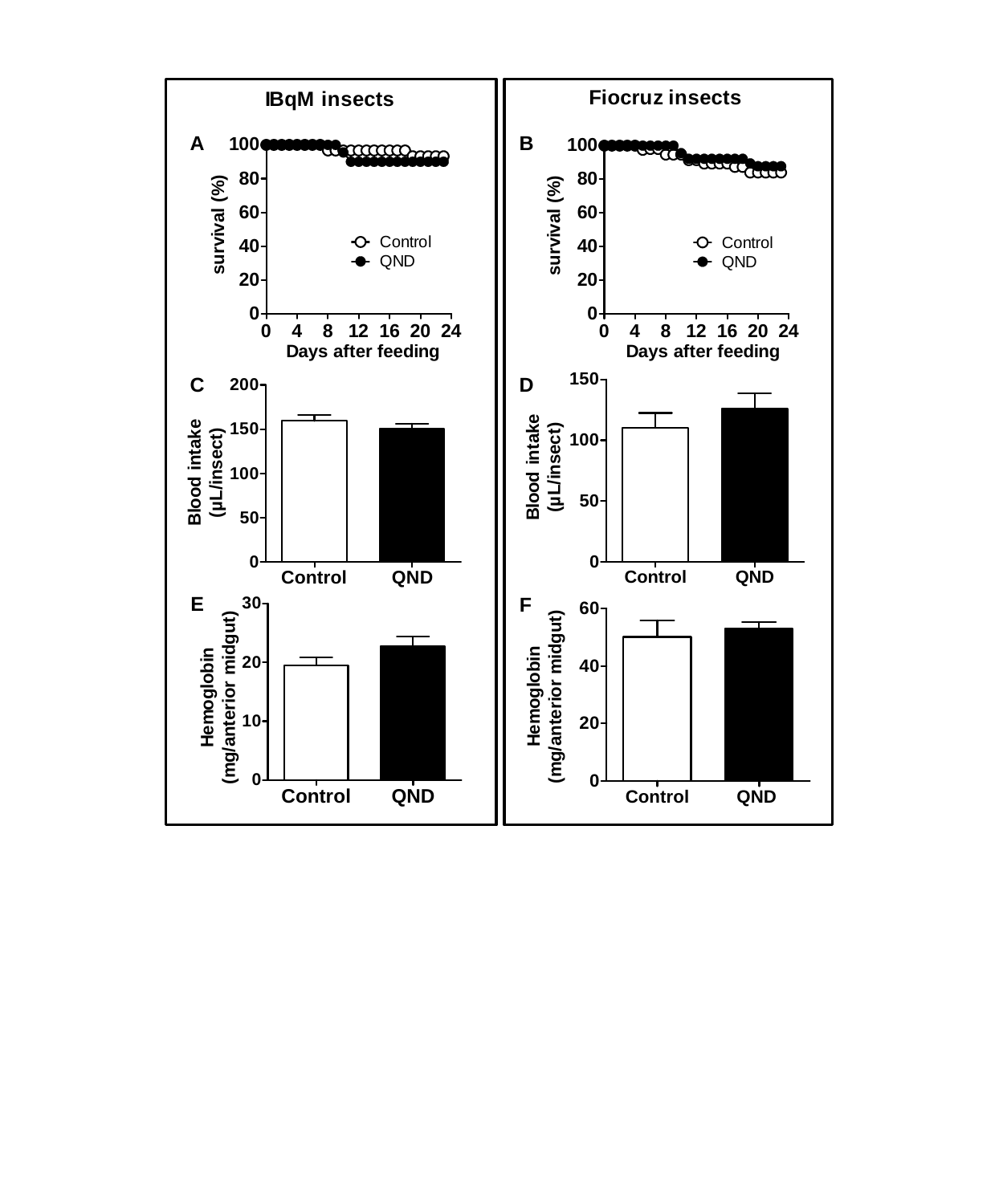

A
B
C
D
E
F

Supplement: S1 Fig — Adult females from different colonies were fed with blood (Control, white circles) or blood supplemented with 100 μM quinidine (QND, black circles). (A and B) Survival of triatomines from IBqM (A, n = 6), or Fiocruz colonies (B, n = 5) were determined along 24 days upon blood meal. (C and D) The average volume (μL) of blood engorged per insect was assessed in insects fed with blood (Control, white bars), or blood supplemented with 100 μM quinidine (QND, black bars), from IBqM (C, n≥41) or Fiocruz (D, n≥21) colonies four days after feeding. Data are expressed as mean ± S.E.M. (E and F) Blood digestion was determined by hemoglobin quantification in the anterior midgut from insects fed with blood (Control, white bars), or blood supplemented with 100 μM quinidine (QND, black bars), from IBqM (E, n≥12) or Fiocruz (F, n = 2) colonies four days after feeding. Data are expressed as mean ± S.E.M. (PPTX) [file pntd.0006661.s001.pptx]

## Slide 1
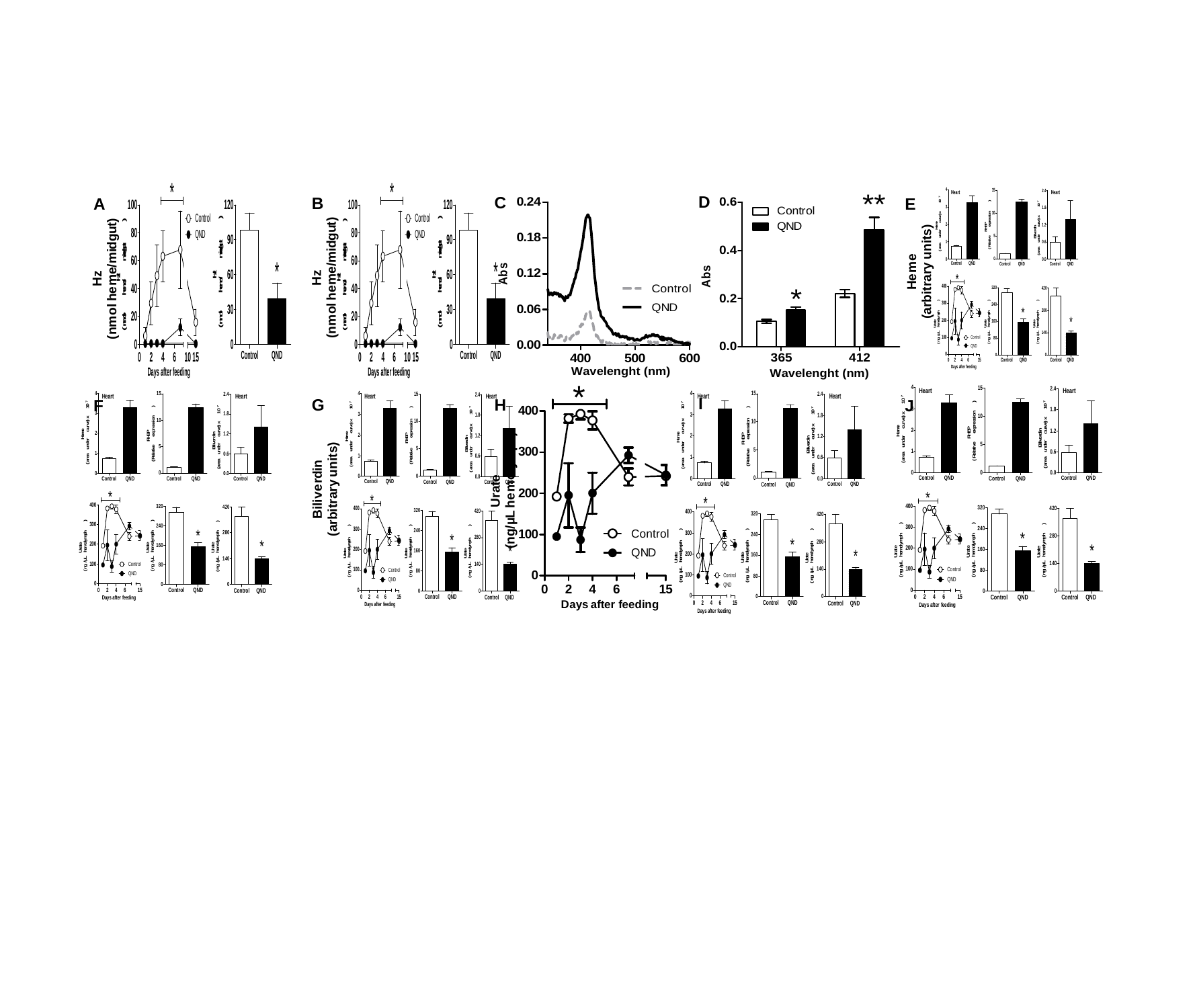

Supplement: S2 Fig — (A) Adult females from Fiocruz colony were fed with blood (Control, white circles, n≥3), or blood supplemented with 100 μM quinidine (QND, black circles, n≥3), and Hz content in posterior midgut was determined along 15 days after blood meal. Comparisons between groups were done by two-way ANOVA and a posteriori Bonferroni’s tests, with *p<0.01 relative to control. (B) Fifth-instar nymphs from IBqM colony were fed with blood (Control, white bar, n = 6) or blood supplemented with 100 μM quinidine (QND, black bar, n = 6), and Hz content in posterior midgut was determined four days after blood meal. Comparisons between groups were done by Mann Whitney’s test, with *p<0.03 relative to control. Data are expressed as mean ± S.E.M. (C and D) Light absorption of hemolymph from adult insects fed with blood (control, white bars, n = 11) or blood supplemented with 100 μM quinidine (QND, black bars, n = 13), from IBqM colony spectrophotometrically determined at 365 nm and 412 nm. Comparisons between groups were done by Student’s t test, with *p<0.05 or **p<0.001 relative to control. Data were expressed as mean ± S.E.M. (E) Total heme levels in hearts from adult insects fed with blood (control, white bars, n = 3) or blood supplemented with 100 μM quinidine (QND, black bars, n = 3), from Fiocruz colony were determined by HPLC four days after blood meal. (F) Relative expression of Rhodnius heme-binding protein (RHBP) in fat bodies of insects fed with blood (control, white bars, n = 2) or blood supplemented with 100 μM quinidine (QND, black bars, n = 2), from Fiocruz colony was assessed by qPCR four days after blood meal. (G) Total biliverdin content in hearts from adult insects fed with blood (control, white bars, n = 3) or blood supplemented with 100 μM quinidine (QND, black bars, n = 3), from Fiocruz colony were determined by HPLC four days after blood meal. (H) Urate levels in the hemolymph from adult insects fed with blood (control, white circles, n≥3) or blood supplemented wit [file pntd.0006661.s002.pptx]

## Slide 1
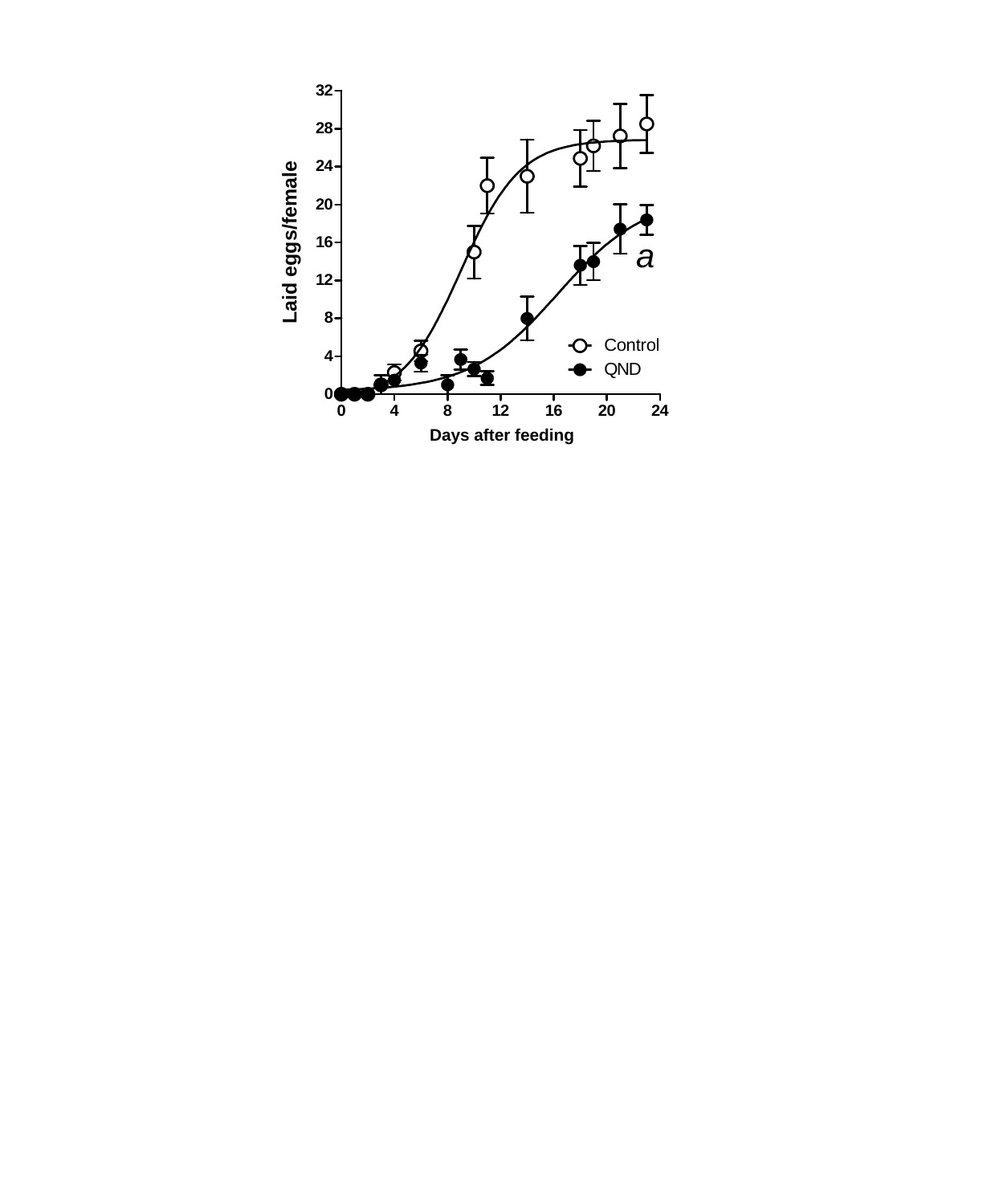

Laid eggs/female
a
Days after feeding

Supplement: S3 Fig — The average number of eggs laid per females from IBqM colony insects fed with blood (control, white circles, n = 13), blood supplemented with 100 μM quinidine (QND, black circles, n = 15) was determined along 24 days upon blood meal. Comparisons between groups were done by two-way ANOVA and a posteriori Bonferroni’s tests, with ap<0.0001 relative to control. Data were expressed as mean ± S.E.M. (PPTX) [file pntd.0006661.s003.pptx]
