# Supplementary material for: Heme crystallization in a Chagas disease vector acts as a redox-protective mechanism to allow insect reproduction and parasite infection
Source: PLoS Negl Trop Dis. 2018 Jul 23;12(7):e0006661. doi: 10.1371/journal.pntd.0006661 (PMC6084092; doi:10.1371/journal.pntd.0006661)
Supplement: S4 Fig — (A) T. cruzi infected adult females from Fiocruz colony were fed with blood (control, white circles, n = 8), blood supplemented with 100 μM quinidine (QND, black circles, n = 8), and total parasite counts in the midgut were carried out after 5 days after blood meal using a Neubauer chamber. The red lines represent medians. (B) T. cruzi infected adult females from Fiocruz colony were fed with blood (control, white circles, n = 21), blood supplemented with 100 μM quinidine (QND, black circles, n = 15), and total parasite counts in the midgut were carried out after 30 days after blood meal using a Neubauer chamber. Comparisons between groups were done by Mann Whitney´s test, with *p<0.005 relative to control. The gray lines represent medians. (C) Total T. cruzi epimastigote counts after incubation for 5 days with 30 μM heme (white bar, n = 6) or 30 μM heme plus 50–100 μM quinidine (black bars, n = 6) was determined by cell counting in a Neubauer chamber. Data are expressed as mean ± S.E.M. Comparisons between groups were done by Kruskal-Wallis, and a posteriori Dunn´s tests, with *p<0.005 relative to control. (D) Total T. cruzi trypomastigote counts after incubation for 2 days with 30 μM heme (white bar, n = 4) or 30 μM heme plus 50–100 μM quinidine (black bars, n = 4) was determined by cell counting in a Neubauer chamber. Data are expressed as mean ± S.E.M. Comparisons between groups were done by Kruskal-Wallis, and a posteriori Dunn´s tests, with *p<0.005 relative to control. (PPTX) [file pntd.0006661.s004.pptx]

## Slide 1
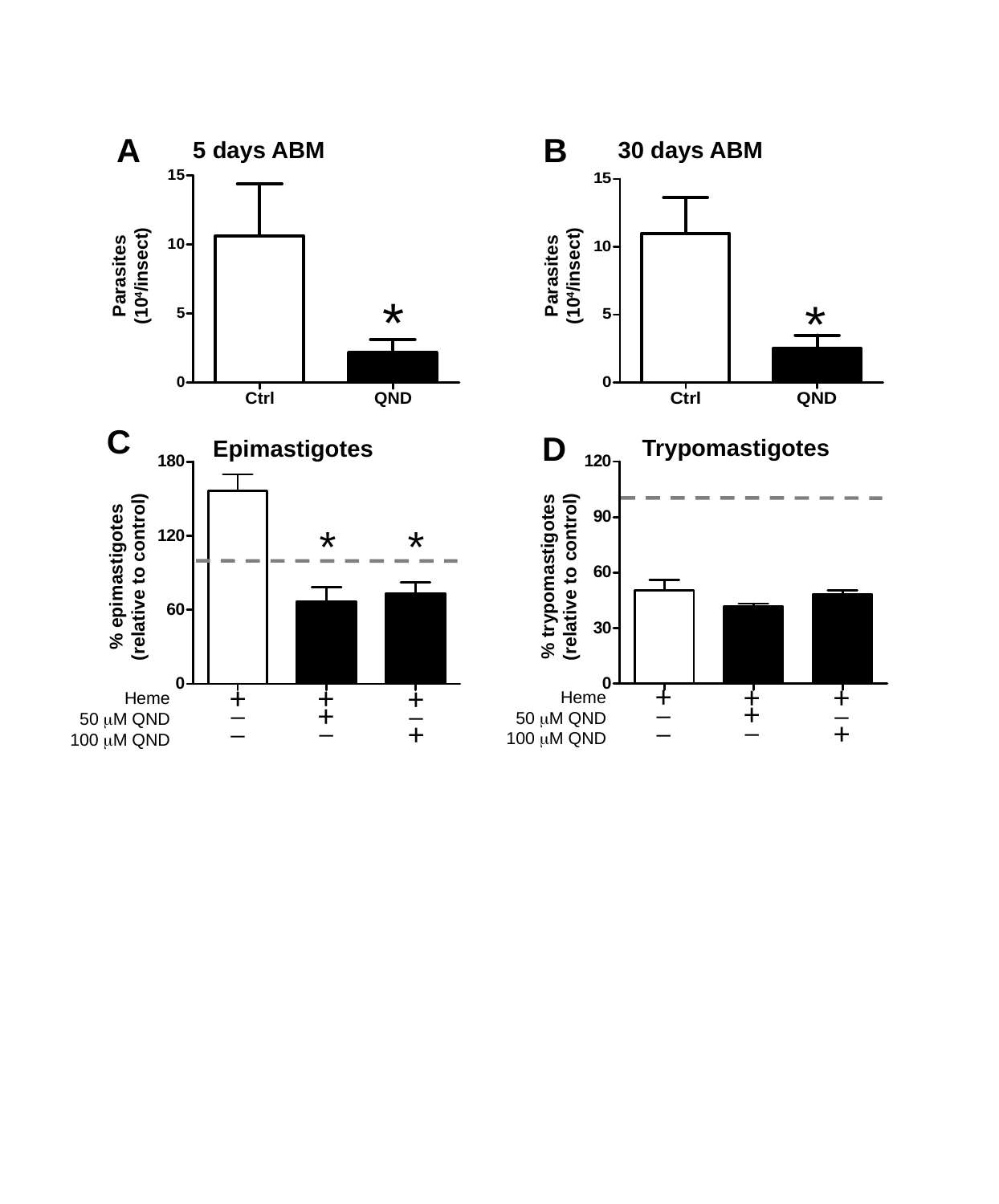

A
B
5 days ABM
30 days ABM
Parasites
(104/insect)
Parasites
(104/insect)
C
D
Trypomastigotes
Epimastigotes
% epimastigotes
(relative to control)
% trypomastigotes
(relative to control)
